# Novel therapeutic strategies targeting resistance mechanisms in hematologic malignancies: from BCL2 inhibition to immunomodulatory approaches

**DOI:** 10.3389/fphar.2025.1742651

**Published:** 2026-01-22

**Authors:** Qianyu Han, Shasha Jiang, Jirui Chen, Lei Xue

**Affiliations:** 1 Department of Thoracic Surgery, The Second Affiliated Hospital of Naval Medical University (Changzheng Hospital), Shanghai, China; 2 The 990th Hospital of the Jointservice Support Force of the PLA, Zhumadian, Henan, China

**Keywords:** bcl2 inhibition, CAR-T cells, drug resistance, hematologic malignancies, immunomodulatorytherapies, venetoclax

## Abstract

**Background:**

Hematologic malignancies, including chronic lymphocytic leukemia (CLL), acute myeloid leukemia (AML), non-Hodgkin lymphoma (NHL), and multiple myeloma (MM), are characterized by high relapse rates due to intrinsic and acquired drug resistance. Resistance mechanisms often involve dysregulation of apoptosis pathways, such as B-cell lymphoma 2 (BCL2) family overexpression, and immune evasion through microenvironment modulation.

**Purpose:**

This review synthesizes recent advances (2020–2025) in therapeutic strategies targeting these mechanisms, focusing on BCL2 inhibition and immunomodulatory approaches to overcome resistance and improve outcomes.

**Methods:**

We systematically reviewed literature from PubMed, Nature, and other databases, emphasizing clinical trials, mechanistic studies, and emerging combinations published between 2020 and 2025. Main Findings: BCL2 inhibitors like venetoclax have achieved high response rates (ORR >70%) in CLL and AML but face resistance *via* MCL1/BCL-XL upregulation. Next-generation agents (e.g., sonrotoclax) and combinations address this. Immunomodulatory therapies, including immunomodulatory imide drugs (IMiDs) and chimeric antigen receptor T-Cell immunotherapy (CAR-T cells), enhance T/NK cell activity, with objective response rate (ORR) up to 90% in relapsed MM. Integrated strategies combining BCL2 inhibition with immunotherapy show synergistic effects, improving progression-free survival (PFS) by 30%–40%.

**Conclusion:**

These strategies represent a paradigm shift toward precision medicine, but challenges like toxicity and biomarker-driven resistance persist. Future directions include AI-guided predictions and novel degraders like proteolysis-targeting chimeras (PROTACs).

## 1 Introduction

### 1.1 Research background and importance

Hematologic malignancies constitute a major global health burden, representing approximately 6.5% of all the incident cancers and 7.2% of cancer-related deaths worldwide (Pan et al., 2025). A predominant challenge in their management is the development of drug resistance, which is the primary driver of poor prognosis in relapsed/refractory (R/R) cases. For instance, The evasion of programmed cell death, or apoptosis, is a hallmark of hematologic cancers (Mirzaei et al., 2017). A pivotal strategy to counter this is BCL2 inhibition, which directly targets the anti-apoptotic machinery upon which many tumor cells depend for survival. Concurrently, immunomodulatory approaches have emerged to restore immune surveillance, which is often disrupted by immunosuppressive tumor microenvironments (TMEs). Recent advances (2020–2025) have shifted from monotherapy to combinations, improving durable remissions.

### 1.2 Key terminology and scope

In this review, drug resistance is defined as the intrinsic (pre-existing) or acquired (treatment-induced) failure of a therapeutic agent to induce a clinical response. Key molecular mechanisms underpinning resistance include the overexpression of BCL2 family anti-apoptotic proteins (e.g., BCL2, myeloid cell leukemia 1 [MCL1], BCL-extra large [BCL-XL]) and the engagement of immune checkpoint pathways (e.g., programmed cell death protein 1/programmed death-ligand 1 [PD-1/PD-L1]).

The scope of this review encompasses novel therapeutic strategies targeting these resistance mechanisms, with a focused discussion on BCL2-targeted therapies and immunomodulatory agents. We will examine their application and efficacy in major hematologic malignancies, including chronic lymphocytic leukemia (CLL), acute myeloid leukemia (AML), non-Hodgkin lymphoma (NHL), and multiple myeloma (MM). Solid tumors are excluded from this discussion. Hodgkin lymphoma, due to its unique tumor microenvironment rich in reactive infiltrating cells and its distinct immune evasion mechanisms, is generally regarded as a distinct immunobiological entity and will not be discussed in this review.

### 1.3 Historical context and milestones

The therapeutic targeting of apoptosis commenced with the development of BH3 mimetics in the early 2000s, a journey that culminated in the landmark U.S. Food and Drug Administration (FDA) approval of venetoclax—a highly selective BCL2 inhibitor—for CLL in 2016, with its indication later extended to AML in 2020(2). In parallel, the era of immunomodulation was heralded by the approval of IMiDs such as lenalidomide for multiple myeloma in 2005, which evolved radically into cellular immunotherapies, exemplified by the approval of axicabtagene ciloleucel, a CD19-directed CAR-T therapy, for NHL in 2017 (Shokati et al., 2025; MacEwan et al., 2021).

The period from 2020 to 2025 has been particularly fruitful, marked by several key milestones. These include the preclinical and clinical emergence of next-generation BCL2 inhibitors like sonrotoclax (BGB-11417), designed to overcome common venetoclax resistance mutations (Liu et al., 2024); a series of approvals for BCMA-targeted CAR-T products and bispecific antibodies for multiple myeloma (Merz et al., 2024); and the rapid clinical translation of integrated strategies that combine BCL2 inhibition with immunomodulatory agents to achieve synergistic efficacy. These advances collectively underscore the ongoing evolution from sequential monotherapies to sophisticated, mechanism-driven combination regimens.

## 2 BCL2 inhibition in overcoming resistance mechanisms

BCL-2 was the first oncogene known to drive tumor growth by suppressing programmed cell death rather than by promoting cellular proliferation (Vaux et al., 1988). In follicular lymphoma, diffuse large B-cell lymphoma (DLBCL) and chronic lymphocytic leukemia (CLL), observed chromosomal translocations juxtapose chromosome 18’s BCL2 gene with chromosome 14’s immunoglobulin heavy chain (IGH) enhancer region, leading to BCL2 overexpression (Barrans et al., 2003). Proteins containing the Bcl-2 homology (BH) domain are collectively termed the Bcl-2 family proteins and play a critical role in mitochondria-mediated apoptosis (Kelly and Strasser, 2011). Based on their function and the number of structural domains, BCL-2 family proteins can be classified into three categories: multi-domain anti-apoptotic proteins (BCL-2, BCL-XL, BCL-w, MCL-1, BCL2A1, BCL-B), multi-domain pro-apoptotic proteins (BAK, BAX, and BOK), and pro-apoptotic proteins containing only the BH3 domain (BID, BIM, BAD, BIK, NOXA, PUMA, BMF, and HRK). The Multi-domain pro-apoptotic protein BAK and BAX can oligomerize within the OMM and form macromolecular pores that subsequently mediate the assembly of the mitochondrial outer membrane permeabilization (MOMP) complex. Cytochrome c and other factors are thereby released from the mitochondrial intermembrane space, initiating apoptosis (Vandenabeele et al., 2023). The anti-apoptotic proteins BCL-2, BCL-XL and MCL-1 possess a hydrophobic groove formed by the BH1-BH3 domains, which can directly bind to the BH3 domains of BAX/BAK to form stable heterodimers, thereby preventing BAX/BAK from converting from “inactive monomers” to “active oligomers” (Vogler et al., 2025). The BH3-only proteins can subdivide into activator and sensitizer proteins. Activator proteins such as BID, BIM, and PUMA bind directly to the BH3 domain-binding groove of BAX/BAK. This binding induces their conformational activation, leading to homo-oligomerization and subsequent pore formation in the Mitochondrial Outer Membrane (MOM) (Moldoveanu et al., 2013; Czabotar et al., 2013). Sensitizer proteins BAD, BIK, BMF, HRK and NOXA interact with anti-apoptotic members and then free “activators” to combine with BAX and BAK. They do not directly combine with BAX and BAK (Kale et al., 2018).

The current primary strategy for targeting BCL-2 in hematologic malignancies involves using agents that structurally mimic BH3-only proteins. These agents competitively bind to the hydrophobic groove of BCL-2, thereby displacing sequestered pro-apoptotic proteins. This displacement reactivates the mitochondria-mediated apoptotic pathway and ultimately induces tumor cell death. The advent of these drugs has marked a paradigm shift in treating hematological malignancies, with venetoclax (VEN) as a representative agent. Venetoclax is a potent and highly selective BCL-2 inhibitor with a highly specific mechanism of action. It precisely targets the BCL-2 protein without acting on BCL-XL, thereby significantly reducing the risk of thrombocytopenia commonly associated with traditional BCL-2/BCL-XL dual-target inhibitors. In the pivotal global Phase III clinical trial VIALE-A, researchers evaluated the efficacy and safety of venetoclax in combination with azacitidine for the treatment of newly diagnosed acute myeloid leukemia (AML) patients who are unsuitable for intensive chemotherapy. The study results showed that the median overall survival (OS) in the venetoclax combination therapy group reached 14.7 months, significantly superior to the 9.6 months in the placebo group (hazard ratio HR = 0.66, *p* < 0.01), indicating that the combination therapy reduced the risk of death by 34% (DiNardo et al., 2020). Based on this groundbreaking achievement, FDA has approved the venetoclax-azacitidine combination regimen for newly diagnosed AML patients aged 75 or older or those with comorbidities that preclude intensive chemotherapy, establishing a new standard treatment option for this patient population. Although venetoclax has achieved remarkable efficacy in the treatment of hematologic malignancies, approximately 30% of patients exhibit primary resistance to venetoclax-based regimens (no initial response), while almost all responding patients ultimately develop secondary resistance and experience disease relapse (DiNardo et al., 2020). Addressing the clinical challenge of resistance necessitates a deeper understanding of how cancers evade cell death despite BCL-2 inhibition.

### 2.1 Common resistance pathways in hematologic malignancies

#### 2.1.1 Compensation and mutations within the BCL-2 protein family

In targeted therapy against the BCL-2 protein for hematological malignancies, the most ommon and direct mechanism of drug resistance stems from compensatory daptations and alterations within the BCL-2 protein family. Among these, the upregulation of other anti-apoptotic proteins represents a major mechanism: when BCL-2 is specifically inhibited, tumor cells frequently upregulate MCL-1 to maintain survival, enabling MCL-1 to continue neutralizing pro-apoptotic proteins such as BIM and BAK, thereby blocking apoptosis (Niu et al., 2016). Similarly, upregulation of BCL-xL can compensate for the loss of BCL-2 function in certain lymphomas and T-cell leukemias; however, inhibiting BCL-xL leads to thrombocytopenia, which has limited the clinical application of corresponding inhibitors (Lin et al., 2016; Dolnikova et al., 2024). Additionally, long-term use of BCL-2 inhibitors like Venetoclax may select for mutations in BCL-2 itself, such as the Gly101Val mutation. This mutation is located within the drug-binding pocket and directly impairs drug-target affinity, thereby conferring resistance (Blombery et al., 2019a). Other mutations such as Asp103Tyr, Phe104Ile, Ala113Gly, etc., are also common in drug-resistant patients (Tausch et al., 2019; Blombery et al., 2019b; Lucas et al., 2020). On the other hand, abnormalities in pro-apoptotic proteins also contribute to resistance: if the key apoptosis executioners BAX or BAK are absent or functionally impaired, then even with effective BCL-2 inhibition, the cell apoptosis program cannot be initiated, ultimately leading to treatment failure (Blombery et al., 2022; Moujalled et al., 2023). Preclinical studies have shown that cells deficient in Activator protein BIM exhibit significantly reduced sensitivity to venetoclax, and the absence of BIM leads to intrinsic resistance (Khaw et al., 2014).

#### 2.1.2 Aberrant activation of upstream signaling pathways

**FIGURE 1:**
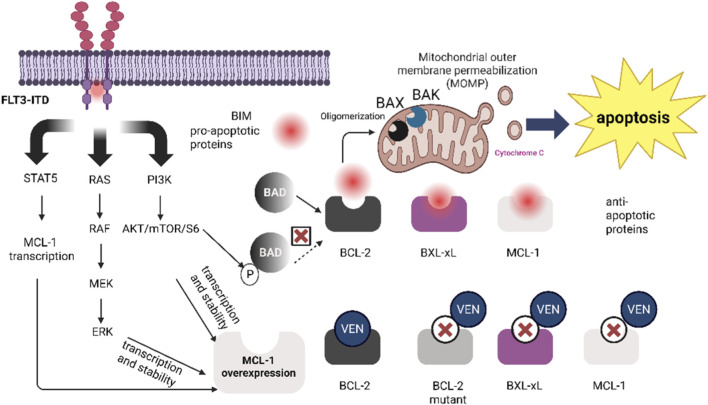
FLT3-ITD signaling-mediated MCL-1 upregulation *via* RAS/MAPK/ERK, PI3K/AKT, and STAT5 pathways drives venetoclax resistance. Anti-apoptotic proteins BCL-2, BCL-XL, and MCL-1 normally bind pro-apoptotic proteins such as BAX and BIM, preventing mitochondrial outer membrane permeabilization (MOMP) and the release of cytochrome c, thereby inhibiting apoptosis. Venetoclax (VEN) binds to BCL-2, freeing pro-apoptotic proteins like BIM and BAX, inducing BAX oligomerization and triggering changes in mitochondrial membrane permeability, ultimately initiating apoptosis. However, the upregulation of non-VEN-binding proteins BCL-XL and MCL-1 (which can be caused by FLT3-ITD mutation-activated pathways such as STAT5, RAS/RAF/MEK/ERK, and PI3K/AKT/mTOR/S6, leading to MCL-1 overexpression through transcriptional and stability regulation), as well as mutations in the BCL-2 binding pocket or BAX protein, can inhibit the apoptotic effect induced by venetoclax, thereby causing drug resistance.

The aberrant activation of upstream cell survival signaling pathways represents an indirect yet crucial drug resistance mechanism. These pathways undermine treatment efficacy by regulating the expression and function of BCL-2 family proteins. For instance, activation of the PI3K/AKT/mTOR pathway not only upregulates MCL-1 expression but also directly stabilizes the MCL-1 protein through AKT-mediated phosphorylation, while simultaneously inactivating the pro-apoptotic protein BAD, thereby disrupting the apoptotic balance (Chong et al., 2025). Similarly, when the MAPK/ERK pathway is activated by mutations in RAS, RAF, etc., it enhances both the transcription and stability of MCL-1 and also leads to BAD inactivation (Scheid et al., 1999; Zhang et al., 2022). Furthermore, in acute myeloid leukemia (AML), the FLT3-ITD mutation persistently activates FLT3 signaling, resulting in concurrent upregulation of MCL-1 and BCL-xL, which significantly reduces the sensitivity of cells carrying this mutation to Venetoclax (Daver et al., 2022). FLT3-ITD signaling can directly upregulate MCL-1 through multiple pathways such as RAS/MAPK/ERK, PI3K/AKT, and STAT5 (particularly STAT5) (Perl, 2019) (Figure 1).

#### 2.1.3 Metabolic reprogramming

Cancer cells can alter their energy metabolism through metabolic reprogramming to adapt to therapeutic stress and develop drug resistance. For instance, while AML cells sensitive to Venetoclax typically rely heavily on oxidative phosphorylation, resistant cells may sustain survival by switching to alternative metabolic modes or enhancing their own oxidative phosphorylation capacity (Chong et al., 2025). Notably, MCL-1, a master regulator of fatty acid oxidation, directly bridges metabolic adaptation with the function of anti-apoptotic proteins (Prew et al., 2022). Additionally, alterations in amino acid metabolism pathways, such as the reprogramming of glutamine metabolism, have also been demonstrated to correlate with acquired resistance to Venetoclax (Chen et al., 2022).

#### 2.1.4 Impact of the tumor microenvironment

**FIGURE 2:**
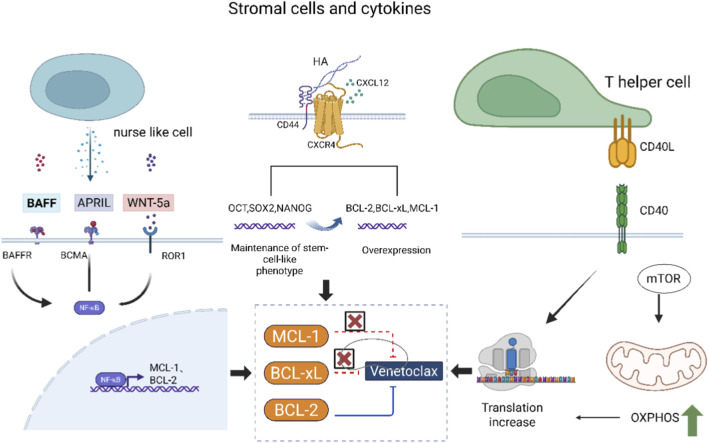
Tumor microenvironment components mediate venetoclax resistance. This diagram outlines three bone marrow microenvironment pathways that reduce Venetoclax efficacy: nurse-like cells secrete BAFF/April/WNT5a, activating NF-κB in CLL cells to upregulate anti-apoptotic proteins; stromal CXCL12/HA drive CXCR4-CD44 complex formation on AML cells, boosting BCL-2 family anti-apoptotic proteins; Helper T cells use CD40L-CD40 signaling to enhance tumor cell translation/OXPHOS, mediating resistance. The “×” denotes Venetoclax’s targeted inhibition of BCL-2, and arrows indicate the direction of signal transduction.

The tumor microenvironment, such as the bone marrow, provides a “sanctuary” for cancer cells. Stromal cells and cytokines(IL-6) within this microenvironment can deliver survival signals that induce the upregulation of anti-apoptotic proteins like MCL-1 and BCL-xL, thereby protecting tumor cells from the cytotoxic effects of Venetoclax (Sadahira et al., 2014; Yu et al., 2021; Ghia et al., 2002). In CLL, “nurse-like cells” not only secrete survival factors (such as BAFF, April, and WNT5a) but also directly physically interact with CLL cells, providing extremely powerful protection against Venetoclax-induced killing (Guo et al., 2022). In acute myeloid leukemia (AML), stromal cell-derived CXCL12 and hyaluronic acid promote the formation of a functional complex between CXCR4 and CD44 on the AML cell surface, thereby enhancing downstream survival signaling and upregulating anti-apoptotic proteins of the BCL-2 family, ultimately leading to resistance against BCL-2 inhibitors. Activated T cells in the tumor microenvironment express CD40L, and the CD40L/CD40 signaling pathway upregulates alternative anti-apoptotic proteins, leading to resistance to Venetoclax (Haselager et al., 2021) (Figure 2).

#### 2.1.5 Lineage specificity and cell differentiation state

In hematologic malignancies, the cell lineage origin and differentiation state of tumor cells collectively determine their inherent dependencies on anti-apoptotic proteins, leading to primary resistance or differential sensitivity to BCL-2 inhibitors. In monocytic-differentiated AML, the leukemic cells typically exhibit constitutively high expression of MCL-1, which confers intrinsic resistance to Venetoclax (Pei et al., 2020). Furthermore, leukemia stem cells (LSCs), which often reside in a quiescent state and rely on unique metabolic pathways and a distinct BCL-2 protein profile (e.g., concurrent dependence on both BCL-2 and MCL-1), are particularly resilient and difficult to eradicate (Kang et al., 2024; Bohler et al., 2021).

#### 2.1.6 Other mechanisms: TP53 loss of function and epigenetic modifications

Beyond the upregulation of alternative anti-apoptotic proteins, resistance to BCL-2 inhibitors like venetoclax can arise through other distinct mechanisms, including functional loss of the TP53 tumor suppressor and epigenetic modifications. TP53 mutations or deletions compromise the intrinsic apoptotic pathway by dampening the transcription of key pro-apoptotic effectors such as BAX, NOXA, and PUMA, thereby reducing sensitivity to venetoclax (Nechiporuk et al., 2019). Alternatively, epigenetic silencing, for instance *via* DNA hypermethylation of promoter regions, can downregulate pro-apoptotic genes like PUMA, offering a potential explanation for acquired resistance even in the absence of clear genetic drivers (Thomalla et al., 2022).

While the elucidated mechanisms underlying resistance to BCL2 inhibitors reveal considerable complexity, these challenges simultaneously illuminate promising directions for next-generation drug development. In response to the pathways described above, researchers are actively pursuing novel strategies and compounds designed to overcome or bypass the limitations of current therapies.

### 2.2 Progression of BCL2 inhibitors development

#### 2.2.1 The emergence of next-generation BCL2 inhibitors

Building upon the success of venetoclax as a benchmark in targeted therapy, several next-generation BCL2 inhibitors with enhanced properties have advanced into clinical development. These novel agents are primarily designed to overcome resistance mechanisms and improve therapeutic efficacy. Among them, sonrotoclax (BGB-11417), developed by BeiGene, demonstrates superior binding affinity to BCL2 compared to venetoclax and retains activity against common resistance mutations such as G101V. It has progressed to phase III trials, showing promising efficacy in combination with the BTK inhibitor zanubrutinib for chronic lymphocytic leukemia (CLL) (Liu et al., 2024; Shadman et al., 2024). Similarly, lisaftoclax (APG-2575) from Ascentage Pharma is structurally optimized for enhanced cellular uptake. In a global, open-label, first-in-human phase I trial, it was evaluated in 52 patients with relapsed/refractory (R/R) chronic lymphocytic leukemia/small lymphocytic lymphoma (CLL/SLL) and other hematologic malignancies, with objectives including safety, tolerability, pharmacokinetics, and preliminary efficacy. The trial demonstrated rapid lymphocyte reduction and an objective response rate (ORR) of 63.6% in efficacy-evaluable R/R CLL/SLL patients, along with a favorable safety profile and no observed clinical tumor lysis syndrome (TLS) (Ailawadhi et al., 2023). Based on these promising results, lisaftoclax has advanced to phase III clinical evaluation for both CLL and acute myeloid leukemia (AML). Additionally, a pipeline of other selective BCL2 inhibitors, including LP-108 and LOXO-338, is under investigation in early-phase trials, broadening the future therapeutic landscape (Kwiatek et al., 2025; Walker et al., 2022).

MCL-1 has emerged as a highly promising therapeutic target due to its key role in mediating resistance to Venetoclax. Preclinical studies of small-molecule MCL-1 inhibitors, exemplified by S63845, have demonstrated potent pro-apoptotic activity. Moreover, their combination with Venetoclax has been shown to effectively reverse resistance driven by MCL-1 overexpression, thereby advancing several candidates into clinical development (Kotschy et al., 2016). However, the clinical translation of MCL-1 inhibitors faces a major obstacle: on-target cardiotoxicity. The clinical development of several MCL-1 inhibitors, including MIK665/S64315 and AMG 397, has been terminated due to cardiotoxicity concerns (Vogler et al., 2025). NA1-115-7 is a natural product isolated from the plant Zygogynum pancheri and represents the first covalent MCL-1 inhibitor. It exhibits no toxicity toward normal blood cells or cardiomyocytes (Daressy et al., 2021) (Table 1).

**TABLE 1:** Representative next-generation BCL-2 inhibitors and MCL-1 inhibitors.

Drug name (inhibitor type)	Developing institution	Key development timeline & highest stage	Relevant clinical trial progress (NCT identifier or status)
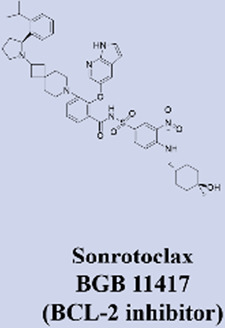	BeiGene	• 2020: First-in-human trial initiated (Phase I). • In October 2025, it received FDA Breakthrough Therapy Designation; • In November 2025, its NDA for MCL (for R/R MCL patients treated with BTK inhibitors) was accepted by the US FDA with priority review status. • Highest Stage: Phase III (for CLL and other indications)	Relevant Clinical Trials: • NCT04277637: A Phase 1a/1b Open-Label Dose Escalation and Expansion Study of Bcl-2 Inhibitor BGB-11417 in Patients With Mature B-Cell Malignancies. • NCT06073821 (CELESTIAL-TNCLL): A Phase 3, Open-Label, Randomized Study of Sonrotoclax (BGB-11417) Plus Zanubrutinib (BGB-3111) Compared With Venetoclax Plus Obinutuzumab in Patients With Previously Untreated Chronic Lymphocytic Leukemia.
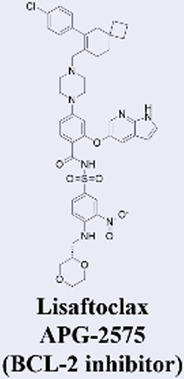	Ascentage Pharma	• In August 2023, it received approval from the U.S. Food and Drug Administration (FDA) to conduct a global registrational Phase III clinical study for the treatment of previously treated patients with CLL/SLL. • In July 2025, NMPA announced that lisaftoclax tablets (APG-2575) have been approved for marketing. • Highest Stage:Approved for Marketing	Relevant Clinical Trials: • NCT04494503: A Phase Ib/II Study of the Safety, Pharmacokinetic, Pharmacodynamic and Efficacy of APG-2575 Single Agent and in Combination With Other Therapeutic Agents in Patients With Relapsed/Refractory CLL/SLL • NCT06641414: A Global Multicenter, Double-blind, Randomized, Registrational Phase 3 Study of Lisaftoclax (APG-2575) in Combination With Azacitidine (AZA) in Patients With Newly Diagnosed Higher Risk Myelodysplastic Syndrome (HR-MDS) (GLORA-4).
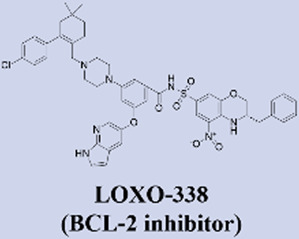	Eli Lilly and Company	• 2020: First-in-human trial initiated (Phase I). • Highest Stage: Phase II (for hematologic malignancies)	Relevant Clinical Trials: • NCT05024045:A Phase 1 Study of Oral LOXO-338, a Selective BCL-2 Inhibitor, in Patients With Advanced Hematologic Malignancies
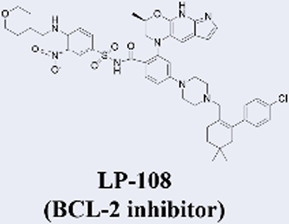	Eli Lilly and Company	• 2022: First-in-human trial initiated (Phase I). • Highest Stage: Phase I (for hematologic malignancies)	Relevant Clinical Trials: • NCT04356846 :A Phase I Study to Evaluate the Safety, Tolerability, Pharmacokinetics, and Preliminary Efficacy of the Oral BCL-2 Inhibitor LP-108 in Patients With Relapsed or Refractory B-cell Lymphoma
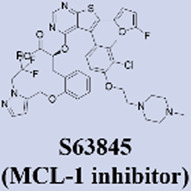	Servier / Novarti	• Highest Stage: Phase I (for hematologic malignancies)	​
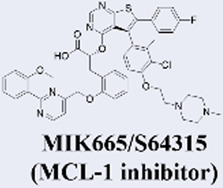	Novartis	• Highest Stage: Phase I (status potentially adjusted/terminated)	Relevant Clinical Trials: • NCT04629443:Phase I/II, International, Multicentre, Open-label, Non-randomised, Non-comparative, Study Evaluating the Safety, Tolerability and Clinical Activity of Intravenously Administered S64315, a Selective Mcl-1 Inhibitor, in Combination With Azacitidine in Patients With Acute Myeloid Leukaemia (AML)
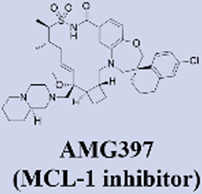	Amgen	• Highest Stage: Phase I (Terminated)	Relevant Clinical Trials: • NCT03465540:A Phase 1 Open-label Study Evaluating the Safety, Tolerability, Pharmacokinetics and Efficacy of AMG 397 in Subjects With Selected Relapsed or Refractory Hematological Malignancies
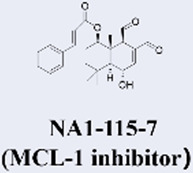	Institut de Chimie des Substances Naturelles	• 2022: First reported as a natural product-derived covalent MCL-1 inhibitor from Zygogynum pancheri in Biomedicine & pharmacotherapy • Highest Stage: Preclinical	​

#### 2.2.2 Exploration of novel targeted strategies

The development of BCL-XL inhibitors has long been hindered by dose-limiting platelet toxicity, a critical challenge that has prompted the emergence of several breakthrough technological strategies in recent years. These innovative approaches, which reconfigure mechanisms of action and optimize delivery pathways, have significantly enhanced the precision and safety of targeted therapies. PROTAC protein degradation technology:DT2216 exemplifies this strategy as a bifunctional molecule that couples a BCL-XL ligand with an E3 ubiquitin ligase ligand to achieve targeted protein degradation. By leveraging the low expression of VHL and other E3 ligases in platelets, it effectively avoids platelet toxicity through tissue-specific degradation (Khan et al., 2019). This approach has now entered Phase I clinical trials (NCT04886622). Antibody-Drug conjugate systems: The ADC candidate ABBV-155 demonstrates a novel targeting strategy by conjugating a BCL-XL inhibitory payload to a B7H3-directed monoclonal antibody. Clinical evaluations have validated this approach’s ability to dissociate on-target antitumor effects from dose-limiting hematological toxicity, achieving preserved therapeutic efficacy without inducing significant thrombocytopenia (Tolcher et al., 2021). It is noteworthy that B7-H3 is overexpressed in solid tumors rather than hematologic malignancies. However, the feasibility of this technological pathway provides important rationale for its potential application in hematologic tumors (requiring a change in target antigen). Nanoparticle drug delivery technology: utilizes dendrimer-based nanocarriers to encapsulate a dual BCL-2/BCL-XL inhibitor(AZD4320), significantly enhancing the therapeutic window through precise modulation of pharmacokinetic properties. This technology improves tumor-specific accumulation and optimizes release kinetics, thereby effectively reducing platelet toxicity. AZD0466 demonstrated significant therapeutic efficacy in both acute myeloid leukemia and T-cell acute lymphoblastic leukemia models (Arulananda et al., 2021). The success of these strategies demonstrates that “inhibition” is not the only approach, while “degradation” and “targeted delivery” represent revolutionary directions for resolving toxicity issues and expanding target space (Table 2).

**TABLE 2:** Novel targeted strategies for BCL-XL inhibitors: PROTAC, ADC, and nanoparticle delivery.

PROTAC	Anti body-drug conjugate	Nano particle drug delivery
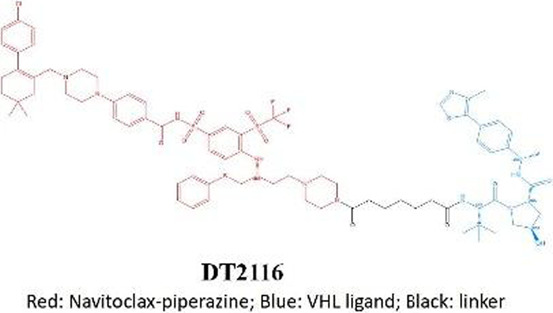	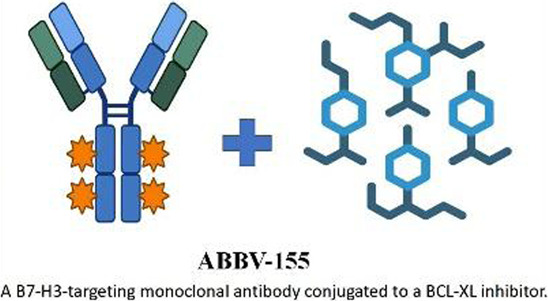	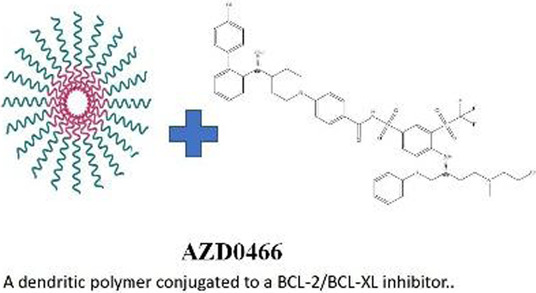

#### 2.2.3 Advancements in combination therapies

In the field of combination therapies involving BCL-2 inhibitors, their application is continuously deepening and optimizing, with combination drug therapies becoming the mainstream standard. Current progress focuses on optimizing combination regimens, exploring new partners, and expanding into earlier lines of treatment. In terms of “chemotherapy-free” regimens, for chronic lymphocytic leukemia (CLL), the fixed-duration regimen of “Venetoclax + anti-CD20 monoclonal antibody (obinutuzumab)” has become one of the first-line standards. Meanwhile, the limited-duration treatment with “Venetoclax + BTK inhibitors (such as ibrutinib, zanubrutinib)” has demonstrated unprecedented deep response rates (such as MRD negativity), making it a preferred option for high-risk patients. In acute myeloid leukemia (AML), the combination of “Venetoclax + hypomethylating agents” has revolutionized the treatment landscape for elderly patients unfit for intensive chemotherapy, becoming a global standard (DiNardo et al., 2020; Al-Sawaf et al., 2020; Tam et al., 2022). Current research is shifting toward applying this combination to patients eligible for intensive chemotherapy, exploring its value in maintenance therapy, and combining it with other targeted drugs (such as IDH1/2 inhibitors, FLT3 inhibitors) in triple regimens.

**FIGURE 3:**
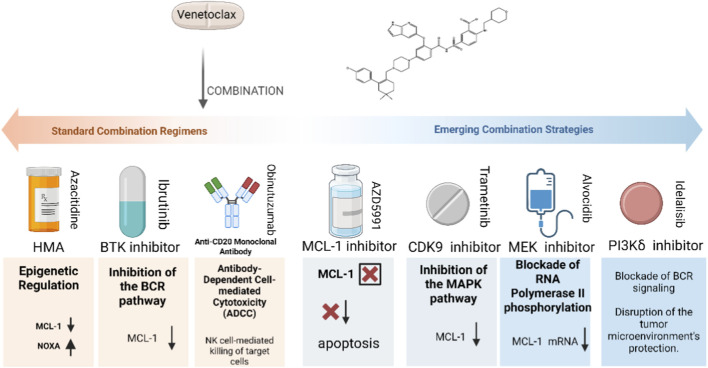
Venetoclax combination therapies: Standard regimens and emerging strategies to enhance efficacy and overcome resistance. This diagram categorizes Venetoclax (a BCL-2 inhibitor) combination strategies into standard regimens and emerging approaches: Standard Combination Regimens (left, orange):Venetoclax + HMA: Epigenetic regulation reduces MCL-1 and increases NOXA (revolutionizing AML treatment for unfit elderly patients); Venetoclax + BTK inhibitor (ibrutinib): Blocks BCR pathway to lower MCL-1 (yields deep responses in high-risk CLL); Venetoclax + anti-CD20 mAb (obinutuzumab): Antibody-dependent cellular cytotoxicity (ADCC) kills tumor cells (first-line CLL standard). Emerging Combination Strategies (right, blue):Venetoclax + MCL-1 inhibitor (AZD5991): Dual inhibition of BCL-2/MCL-1 induces apoptosis (targets MCL-1-driven resistance); Venetoclax + CDK9/MEK inhibitors: Targets pathways regulating MCL-1 (reduces MCL-1 expression *via* mRNA/protein downregulation); Venetoclax + PI3Kδ inhibitor (idelalisib): Blocks BCR signaling to disrupt tumor microenvironment protection. “×” denotes target inhibition.

To overcome resistance, novel combination strategies are continuously emerging. Among them, the most prominent is “BCL-2 + MCL-1 dual inhibition,” which combines Venetoclax with MCL-1 inhibitors (such as S63845) to simultaneously block two major survival dependencies. Although this approach has proven effective in preclinical models for overcoming resistance driven by MCL-1 upregulation, and several combination regimens have entered Phase I/II clinical trials (particularly in AML and MM), the overlapping myelosuppressive toxicity (such as neutropenia) remains a significant challenge, making the identification of a safe window critical (Algarín et al., 2021). Additionally, combinations with inhibitors of other pathways show promise. For example, combining with MEK inhibitors to target the RAS/MAPK pathway, which drives MCL-1 expression; combining with CDK9 inhibitors to rapidly downregulate MCL-1 mRNA and protein levels; and in lymphoma and CLL, combining with PI3Kδ or SYK inhibitors to block the BCR signaling pathway, thereby weakening the protective effects of the tumor microenvironment (Zhang et al., 2022; Jonas et al., 2023; Elias et al., 2022) (Figure 3).

#### 2.2.4 Clinical application of precision medicine

Currently, the application of biomarkers and functional testing enables the implementation of precision medicine strategies targeting BCL-2 inhibitors in hematologic malignancies. In the realm of predictive biomarkers, established positive indicators include the t (Vogler et al., 2025; Kale et al., 2018) translocation and high BCL2 expression, whereas negative indicators associated with poorer outcomes encompass TP53 mutations, the BCL2 G101V resistance mutation, and upregulation of other anti-apoptotic proteins such as MCL1 or BCL-XL. The t (Vogler et al., 2025; Kale et al., 2018) chromosomal translocation is the strongest predictive biomarker for sensitivity to Venetoclax in patients with multiple myeloma (MM). Emerging functional assays such as BH3 profiling directly evaluate tumor cells’ “apoptotic priming” and functional dependencies on specific anti-apoptotic proteins, enabling the identification of resistance mechanisms—such as detecting cancer cells switching dependency from BCL-2 to MCL-1—and guiding precise matching with corresponding BH3 mimetics (Jacquier et al., 2025; Smith et al., 2020). Concurrently, dynamic monitoring of resistance mutations like BCL2 G101V through ddPCR/NGS has become standard practice during treatment, allowing for timely therapeutic strategy adjustments (Khalsa et al., 2023).

## 3 Immunomodulatory approaches to reverse immune evasion

### 3.1 Mechanisms of immune resistance

Immune resistance in hematologic malignancies is a complex adaptive process wherein tumor cells evolve multiple strategies to evade immune surveillance, especially under the selective pressure of therapies such as allogeneic hematopoietic cell transplantation (allo-HCT) and T-cell-targeting immunotherapies (Arandi and Dehghani, 2023).

A key escape mechanism is the disruption of antigen presentation, which renders malignant cells functionally “invisible” to effector T cells (Craddock and Friedberg, 2021). This can result from irreversible genomic events (e.g., copy-neutral loss of heterozygosity leading to loss of an HLA haplotype) or from reversible epigenetic downregulation of HLA class II molecules and their regulator CIITA (Chergui and Reagan, 2023). Intrinsic defects in antigen-processing machinery, often linked to TP53 mutations or MDM2 overexpression, further restrict the antigen repertoire presented to T cells (Bruzzese et al., 2025).

Tumor cells also actively establish an immunosuppressive milieu by upregulating inhibitory ligands such as PD-L1, B7-H3 and PVRL2 (Tsumura et al., 2023). These ligands engage receptors like PD-1, TIM-3 and TIGIT on tumor-infiltrating T cells, creating a potent inhibitory circuit. Oncogenic drivers can fuel this process; for example, IDH1/2 mutations produce D-2-hydroxyglutarate that directly impairs T-cell function, while TP53 abnormalities are associated with expansion of regulatory T cells and myeloid-derived suppressor cells (Tang et al., 2023; Mountjoy et al., 2020). Consequently, T cells undergo profound dysfunction, manifesting as exhaustion or senescence (Noh et al., 2020).

Further compounding this immune suppression is the metabolic rewiring of the tumor microenvironment. A fierce competition for nutrients and the accumulation of waste products, such as lactic acid from glycolytic metabolism, create a hostile milieu that directly impairs T-cell metabolism, proliferation, and cytokine production (Slavin, 2023). This metabolic sabotage is a key driver of relapse post-transplant. Additionally, oxidative stress induced by conditioning regimens and graft-versus-host disease can lead to oxidative DNA damage in reconstituting T cells, predisposing them to exhaustion and diminishing their anti-leukemic capacity (Liu et al., 2021). Finally, a direct and potent form of resistance to targeted immunotherapies, such as CAR-T cells and bispecific antibodies, is the simple loss of the target antigen on the tumor cell surface, as commonly seen with CD19 in B-cell malignancies (Tatake et al., 2024). In aggregate, these interconnected mechanisms--spanning impaired antigen recognition, active immune suppression, T-cell functional decay, and metabolic barriers--form a formidable biological foundation for treatment failure and disease relapse.

### 3.2 Recent immunomodulatory innovations

**FIGURE 4:**
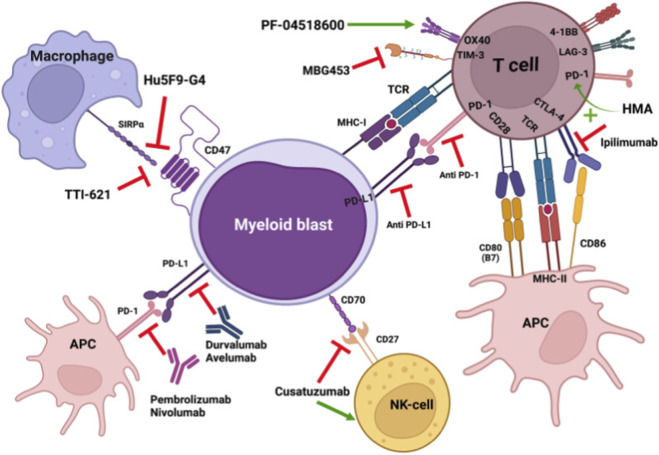
Immunomodulatory approaches to reverse immune evasion. Boosting immune response through blocking inhibitory pathways and activating immune cells. HMA: hypomethylating agents; APC: antigen-presenting cell; MHC: Major histocompatibility complex. Copy right from J Hematol Oncol. 2022 Aug 31; 15(1):124. (Mohty et al., 2022).

Hematologic malignancies have emerged as a proving ground for cancer immunotherapy, leveraging the immune system to target and eliminate malignant cells. The unique biology of blood cancers, coupled with accessible tumor antigens, has facilitated the development of numerous immunotherapeutic approaches (Figure 4).

#### 3.2.1 Immunomodulatory imide drugs (IMiDs)

IMiDs, including lenalidomide, pomalidomide, and next-generation agents such as iberdomide and CC-92480, function through binding to cereblon (CRBN), the substrate receptor of the CRL4 E3 ubiquitin ligase complex. This interaction induces ubiquitination and subsequent degradation of specific substrates, particularly Ikaros family zinc finger proteins 1 and 3 (IKZF1 and IKZF3), and casein kinase 1 alpha. The degradation of these proteins leads to complex downstream effects including activation of pro-apoptotic pathways *via* GPR68/calcium/calpain signaling and inhibition of the RCAN1/calcineurin pro-survival pathway. Additionally, IMiDs enhance natural killer (NK) cell-mediated antibody-dependent cellular cytotoxicity (ADCC) and modulate various cytokine pathways within the tumor microenvironment.

In multiple myeloma, IMiDs have demonstrated remarkable efficacy, both as monotherapy and in combination regimens. When combined with CD38-targeting monoclonal antibodies or proteasome inhibitors, IMiDs achieve overall response rates (ORR) of 50%–70% in relapsed/refractory multiple myeloma (RRMM) (Mountjoy et al., 2020). Beyond multiple myeloma, lenalidomide has established efficacy in myelodysplastic syndromes with deletion 5q, where it reduces transfusion dependence and promotes hematologic improvement (Gardner et al., 2024; Park et al., 2021). Despite their clinical success, IMiDs face several challenges. Resistance frequently develops through CRBN downregulation or mutations, limiting long-term efficacy. Significant toxicities include myelosuppression, increased risk of venous thromboembolism, and secondary primary malignancies, particularly acute lymphoblastic leukemia and squamous cell carcinoma of the skin. Drug interactions present additional challenges, as PPAR agonists can downregulate CRBN expression, potentially compromising IMiD efficacy (Sha et al., 2022)).

#### 3.2.2 Chimeric antigen receptor (CAR)-T cell therapy

CAR-T cell therapy represents a paradigm shift in cancer treatment, involving genetic modification of patient-derived T cells to express chimeric antigen receptors that recognize specific tumor antigens (Mougiakakos et al., 2025). First-generation CARs incorporated CD3ζ signaling domains, while contemporary constructs include multiple costimulatory domains (CD28, 4-1BB) to enhance persistence and efficacy (Kong et al., 2024; Jogalekar et al., 2022). Recent innovations include “armored” CARs secreting cytokines or ligands to overcome immunosuppressive microenvironments, allogeneic “off-the-shelf” CAR products, and multi-targeting CARs to prevent antigen escape (Xia et al., 2024; Dabas and Danda, 2023).

CD19-directed CAR-T therapies have demonstrated remarkable efficacy in B-cell malignancies. In diffuse large B-cell lymphoma, axicabtagene ciloleucel achieves complete response rates of 40%–50% in patients refractory to conventional therapies. Similarly, in multiple myeloma, BCMA-targeting CAR-T products such as idecabtagene vicleucel show ORR exceeding 80%, though median progression-free survival remains limited to approximately 12 months. Recently, long-term 5-year follow-up data from the CARTITUDE-1 clinical trial of another BCMA-targeting CAR-T product, Ciltacabtagene Autoleucel, were also presented at the ASCO annual meeting. The results showed that among patients who had undergone extensive prior treatment, this regimen achieved a 5-year progression-free survival rate of 33% (without requiring additional interventions during this period) and a median overall survival of 60.7 months (95% confidence interval: 41.9 months to not reached) (Merz et al., 2024). Emerging targets including GPRC5D and FcRH5 show promise in early-phase trials for multiple myeloma. Cytokine release syndrome (CRS) and immune effector cell-associated neurotoxicity syndrome (ICANS) represent significant challenges, occurring in up to 80% and 40% of patients, respectively (Grant et al., 2022; Han et al., 2024; Hu and Dunbar, 2024). Management algorithms incorporating tocilizumab (IL-6 receptor antagonist) and corticosteroids have improved safety profiles. Resistance mechanisms include antigen loss or modulation, T-cell exhaustion characterized by upregulation of inhibitory receptors, and immunosuppressive tumor microenvironments rich in regulatory T cells and myeloid-derived suppressor cells (Albarrán et al., 2024). Manufacturing complexities and specialized care requirements further limit accessibility, particularly in resource-limited settings.

#### 3.2.3 Bispecific T-cell engagers (BiTEs)

BiTEs represent a novel class of bispecific antibodies that simultaneously engage CD3 on T cells and tumor-associated antigens, facilitating T-cell activation and tumor cell lysis independent of MHC restriction (Verma and Sharma, 2024; Jamali et al., 2025). Structural innovations include extended half-life formats through Fc domain incorporation, optimized binding affinities to prevent T-cell exhaustion, and multi-specific constructs targeting multiple tumor antigens simultaneously to reduce antigen escape (Hays, 2022; Gerber et al., 2025). CD20-directed BiTEs such as glofitamab and mosunetuzumab demonstrate ORR of 70% and complete response rates of 40% in relapsed/refractory non-Hodgkin lymphoma. In multiple myeloma, BCMA-targeting teclistamab achieves ORR of 64% with durable responses observed in heavily pretreated populations. The recent emergence of GPRC5D-targeting bispecific antibodies shows additional promise for multiple myeloma patients previously exposed to BCMA-directed therapies (Merz et al., 2024). CRS represents the most frequent toxicity, though typically less severe than observed with CAR-T therapies. ICANS, cytopenias, and infection risks require careful monitoring (Edeline et al., 2021). Most current BiTE constructs require continuous intravenous infusion, though subcutaneous formulations with extended half-lives are under development to improve convenience and outpatient management (Morviducci et al., 2025).

#### 3.2.4 NK cell therapies

NK cells mediate antitumor activity through multiple mechanisms, including direct cytotoxicity, ADCC, and cytokine secretion. Current engineering approaches include CAR-NK constructs, cytokine-induced memory-like NK cells with enhanced persistence, and iPSC-derived NK products for standardized manufacturing (Merino et al., 2023). Genetic modifications to enhance homing, persistence, and resistance to immunosuppression are actively being explored.

CAR-NK cells targeting CD19 demonstrate promising efficacy in B-cell malignancies with response rates comparable to early CAR-T experiences, while notably lacking significant CRS or ICANS (Baghery et al., 2022). Cord blood-derived CAR-NK cells show response rates exceeding 70% in relapsed/refractory CD19-positive malignancies. Tri-specific killer engagers (TriKEs) incorporating IL-15 stimulation demonstrate enhanced NK persistence and cytotoxicity in early clinical trials (Jørgensen et al., 2025). The limited *in vivo* persistence of NK cells remains a significant hurdle, with most adoptively transferred NK cells detectable for less than 2 weeks. The immunosuppressive tumor microenvironment, particularly TGF-β secretion, inhibits NK function. Manufacturing standardization across different NK sources requires further development for widespread clinical implementation (Fetzko et al., 2023; Rana et al., 2022). The “off-the-shelf” potential of NK cell therapies offers economic advantages over patient-specific approaches. Their favorable safety profile, particularly the absence of GVHD and reduced risk of CRS/ICANS, makes them particularly suitable for elderly and pediatric populations (Lamb et al., 2021).

Emerging targets including CD47, TIM-3, and GPRC5D show promise in early clinical development. Rational combination strategies integrating CAR-T or BiTEs with IMiDs, checkpoint inhibitors, or tumor microenvironment modulators aim to overcome resistance mechanisms (Garfall, 2024; Rallis et al., 2021). Sequential therapy approaches, such as BiTE exposure followed by CAR-T infusion, may enhance T-cell fitness and antitumor efficacy.

Next-generation cellular therapies incorporating safety switches, inducible cytokine expression, and resistance to inhibitory signals are in clinical development. Universal allogeneic products from healthy donors or iPSC sources aim to overcome manufacturing limitations. Gene editing technologies facilitate enhanced persistence and function while reducing alloreactivity. Robust biomarkers predicting response and toxicity are critically needed (Rallis et al., 2021). Tumor-intrinsic factors including antigen density and mutational burden, along with host factors such as immune repertoire and inflammatory status, may guide patient selection and therapy personalization (Rana et al., 2022). Minimal residual disease monitoring integrated with immunotherapy administration represents a promising approach for preventing overt relapse.

## 4 Integrated strategies: Combining BCL2 inhibition and immunomodulation

### 4.1 Synergistic mechanisms

The treatment paradigm for hematologic malignancies is undergoing a fundamental shift—moving away from monotherapies that solely induce direct tumor cell death toward integrated strategies that concurrently engage the immune system. One of the most promising approaches is the combination of BCL2 inhibition with immunomodulatory agents. The synergy between BCL2 inhibitors (such as Venetoclax) and immunotherapies represents a cutting-edge strategy to overcome drug resistance and enhance therapeutic efficacy in blood cancers. At the core of this combined approach lies the concept of leveraging immune-mediated attacks to bypass apoptotic resistance pathways in tumor cells, while simultaneously using BCL2 inhibitors to undermine the survival defenses of malignant cells, thereby achieving synergistic tumor eradication.

The synergistic mechanism begins with BCL-2 inhibitor-induced immunogenic cell death, wherein the release of tumor-associated antigens and damage-associated molecular patterns effectively initiates dendritic cell-mediated T cell priming, laying the foundation for adaptive immune responses. Genetic knockout or pharmacological inhibition of BCL2 with venetoclax or navitoclax was shown to significantly enhance the antigen presentation capacity of conventional type 1 dendritic cells (cDC1s) (Zhao et al., 2023). BCL-2 overexpression in hematologic malignancies, such as CLL and AML, not only confers a cell-intrinsic survival advantage but also erects a formidable inhibitory barrier against cytotoxic T cells. Subsequently, the rapid and profound clearance of tumor burden by BCL-2 inhibitors directly dismantles the physical and functional immunosuppressive barriers established by tumors. At the immune effector stage, the combination strategy further exerts synergistic effects through direct modulation of immune cells: BCL-2 inhibition can eliminate chronically stimulated lymphocytes, thereby reducing T-cell exhaustion and potentially rejuvenating the immune response against tumors (Wei et al., 2023). Meanwhile, Venetoclax can also increase the production of reactive oxygen species (ROS), thereby enhancing T cell-mediated anti-leukemic activity (Lee et al., 2021). Venetoclax augments the efficacy of anti-CD20 immunotherapy by enhancing antigen-specific T-cell responses, through the restoration and maintenance of activatable naive and effector CD8^+^ T cells at the tumor site (Melchor et al., 2023). Venetoclax triggers the irreversible mitochondrial apoptosis pathway by inhibiting BCL-2. The accompanying structural changes in the cell membrane, particularly the externalization of, underlie the enhanced antibody-dependent cellular phagocytosis and complement-dependent cytotoxicity observed when combined with rituximab or Anti-CD47 immunotherapy in the treatment of hematologic malignancies (Li et al., 2022). Furthermore, the combination of BCL-2 inhibitors and PD-1/PD-L1 inhibitors creates a mechanistically complementary approach: BCL-2 inhibitors drive type I interferon production and innate immune responses by activating the cGAS-STING signaling pathway, while PD-1/PD-L1 inhibitors block immunosuppressive signals, thereby reversing T-cell functional suppression (Zhang et al., 2025). Through this synergistic action, the combination is theoretically capable of significantly enhancing antitumor immunity, achieving a “1 + 1 > 2” therapeutic effect. Ultimately, these changes collectively drive a fundamental remodeling of the tumor microenvironment, reversing it from an immunosuppressive “cold” state to an immune-infiltrated “hot” state. This transformation not only enhances the efficacy of immune attacks but, more critically, overcomes BCL-2 inhibitor resistance mediated by the tumor microenvironment, thereby establishing a self-reinforcing therapeutic cycle that enables sustained disease control.

### 4.2 Recent clinical advances

These compelling mechanistic rationale have rapidly translated into promising clinical investigations. Emerging clinical evidence supports the translational relevance of these findings. For instance, analysis of peripheral blood from acute myeloid leukemia (AML) patients treated with venetoclax revealed clear activation of circulating cDC1s, evidenced by upregulated surface expression of CD86, CCR7, and HLA-DQ (Zhao et al., 2023). In the absence of cDC1 (e.g., in Batf3-KO mice) or T cells, BCL2 inhibitors are unable to suppress tumor growth. This provides direct human evidence for the immunostimulatory effects of BCL2 inhibition and has spurred numerous clinical trials exploring combinations of venetoclax with PD-1/PD-L1 inhibitors in various lymphomas and leukemias (Table 3).

**TABLE 3:** Overview of venetoclax combined with PD-1/PD-L1 inhibitors under clinical investigation for hematological malignancies.

Clinicaltrials. Gov identifier	Study name	Status	Interventions	Drug regimen	Study population	Study phase	Primary outcome measures	Enrollment	Year of initiation
NCT05388006	Acalabrutinib, venetoclax and durvalumab for richter transformation	Recruiting	Acalabrutinib; durvalumab; venetoclax; biospecimen collection; imaging	BTKi + anti-PD-L1 + BCL-2i	Patients with richter transformation	Phase 2	Progression-free survival at 6 months	27	2023
NCT04277442	Nivolumab + decitabine + venetoclax in TP53-mutated AML	Completed	Decitabine; nivolumab; venetoclax	HMA + anti-PD-1 + BCL-2i	Newly diagnosed TP53-mutated AML patients	Phase 1	Incidence of adverse events; feasibility (patients completing three cycles); treatment response	1	2020
NCT03969446	Pembrolizumab + decitabine ± venetoclax in AML/MDS	Recruiting	Decitabine; pembrolizumab; venetoclax	HMA + anti-PD-1 ± BCL-2i	Patients with newly diagnosed, recurrent, or refractory AML/MDS	Phase 1b	Incidence of adverse events; maximum tolerated dose; treatment response rate	54	2020
NCT03390296	OX40, venetoclax, avelumab, glasdegib, gemtuzumab ozogamicin, azacitidine in R/R AML	Completed	PF-04518600 (Anti-OX40); avelumab; azacitidine; gemtuzumab ozogamicin; glasdegib; venetoclax	Immune agonist + anti-PD-L1 + HMA + ADC + hedgehog inhibitor + BCL-2i	Patients with relapsed or refractory AML	Phase 1/2	Rate of complete response (CR + CRp + CRi) within 3 months	50	2017

A multi-arm phase Ib/II clinical trial in relapsed/refractory acute myeloid leukemia (R/R AML) evaluated various immunotherapy combinations, demonstrating the potential of venetoclax combined with the PD-L1 inhibitor avelumab. Although the overall response rate was modest, deep and durable remissions (>15 months) were observed in specific patient subsets (e.g., those with extramedullary disease), providing valuable insights for immunotherapy applications in AML. Furthermore, the azacitidine + venetoclax + gemtuzumab ozogamicin regimen achieved a 50% CR/CRi rate in venetoclax-naive patients with no dose-limiting toxicities observed and favorable tolerability, highlighting the significant potential of combining venetoclax with PD-L1 inhibitors (Short et al., 2022). Furthermore, it is important to acknowledge that most combinations of venetoclax with checkpoint inhibitors remain in early-phase clinical investigation, and mature clinical data regarding their long-term efficacy and safety are still limited.

Venetoclax + anti-CD20 monoclonal antibody (Rituximab and Obinutuzumab) has become one of the standard treatments for R/R CLL and has demonstrated significant efficacy in high-risk patients with untreated CLL, MCL, and DLBCL. Early-phase trials in suggest that adding venetoclax to R-CHOP chemotherapy may be particularly beneficial for patients with the double-expressor phenotype (Morschhauser et al., 2021). With long-term follow-up in the MURANO study, fixed-duration VenR in R/R CLL yielded a profound survival benefit (5-year PFS and OS rates of 53.6 months and 82.1%, respectively) and a high rate of uMRD (62%), underpinning the long-term efficacy of this time-limited approach (Kater et al., 2020). For previously untreated patients with chronic lymphocytic leukemia (CLL) and comorbidities, the phase III CLL14 trial demonstrated that fixed-duration venetoclax plus obinutuzumab (Ven-Obi), compared with chlorambucil plus obinutuzumab (Clb-Obi), significantly improved progression-free survival (6-year PFS rate: 76.2% vs. 36.4%; HR 0.31–0.40), induced a higher rate of undetectable minimal residual disease (uMRD <10^−6^ in 40% of patients), and led to early improvement in quality of life, thereby establishing its position as a standard first-line treatment (Al-Sawaf et al., 2024; Al-Sawaf et al., 2021).

Beyond traditional anti-CD20 monoclonal antibodies, next-generation CD20 bispecific antibodies provide a more compelling mechanistic rationale for combination therapy with BCL2 inhibitors. By simultaneously targeting CD20 (on tumor cells) and CD3 (on T cells), these agents directly redirect and activate endogenous T cells to mediate potent tumor cell killing. For example, the subcutaneously administered CD3×CD20 bispecific antibody epcoritamab has been shown in preclinical studies to effectively induce lysis of primary chronic lymphocytic leukemia (CLL) cells. *In vitro* models further demonstrate that the combination of epcoritamab and venetoclax exhibits synergistic antitumor activity, with cytotoxicity significantly surpassing that of either agent alone. In terms of clinical research, the combination strategies of epcoritamab have shown progress across different indications. Recently, results from the phase 1b/2 EPCORE NHL-2 study demonstrated that a fixed-duration regimen of epcoritamab combined with rituximab and lenalidomide (treatment up to 2 years) achieved deep and durable efficacy in patients with relapsed/refractory follicular lymphoma: after a median follow-up of over 28 months, the overall response rate was as high as 96%, the complete response rate reached 88%, and 86% of evaluable patients achieved minimal residual disease negativity. Concurrently, clinical trials evaluating the combination of epcoritamab with BTK inhibitors or venetoclax (such as the EPCORE CLL-1 trial, NCT04623541) are also underway. Furthermore, other CD20 bispecific antibodies, such as glofitamab and mosunetuzumab, have also demonstrated notable clinical activity in lymphoma treatment, making their combination with BCL2 inhibitors an important direction for future research.

Venetoclax in combination with IMiDs is feasible and effective in the treatment of relapsed/refractory diffuse large B-cell lymphoma and mantle cell lymphoma. In relapsed/refractory diffuse large B-cell lymphoma (DLBCL), the ViPOR regimen (venetoclax, ibrutinib, prednisone, obinutuzumab, and lenalidomide) achieved an objective response rate of 54% and a complete response rate of 38%, with 2-year progression-free and overall survival rates of 34% and 36%, respectively, particularly in non-germinal center B-cell-like subtypes (Melani et al., 2024). In mantle cell lymphoma, MRD-driven venetoclax plus lenalidomide and rituximab (venetoclax-R2) yielded an overall response rate of 63%, which remained at 40% in patients who had failed prior BTK inhibitor therapy, with median progression-free survival of 21 months. TP53 mutation was associated with inferior outcomes in this cohort (Kumar et al., 2017). IMiDs are commonly used in the treatment of multiple myeloma. An initial phase II study investigating the efficacy of venetocla 9x in combination with pomalidomide and dexamethasone (VenPd) for lenalidomide-refractory multiple myeloma showed promising activity, with an overall response rate of 63% and a median progression-free survival of 10.5 months in eight enrolled patients. However, the study was terminated early due to a high incidence of grade ≥3 adverse events, particularly neutropenia, underscoring both the potential and notable safety concerns associated with this regimen (Gasparetto et al., 2021).

Current clinical evidence supporting the “Venetoclax + CAR-T” combination therapy remains limited, primarily derived from preclinical studies and retrospective analyses. Preclinical investigations by Su et al. demonstrated that venetoclax treatment significantly upregulates ITGB2 (integrin β2) expression on Nalm6 cells, which markedly enhances the cytotoxic activity of CAR-T cells against these target cells. Clinically, in a retrospective study by Zhu et al. (2023) involving 14 patients with relapsed/refractory diffuse large B-cell lymphoma who had failed prior CAR-T therapy, venetoclax-based salvage regimens achieved an overall response rate of 42.9% (6/14) and a complete response rate of 21.4% (3/14). These findings collectively provide important mechanistic insights and preliminary clinical support for the potential synergistic efficacy of this combination strategy (Su et al., 2025; Zhu et al., 2023). Clinical exploration of the “Venetoclax + CAR-T” combination therapy is rapidly advancing in the field of B-cell malignancies, particularly showing promising synergistic therapeutic potential in acute lymphoblastic leukemia. Multiple interventional studies led by leading hematology centers in China have entered the recruitment phase: In Philadelphia chromosome-negative B-ALL, researchers are evaluating the efficacy and safety of chemotherapy combined with CAR-T and Venetoclax (NCT06481241); while in Philadelphia chromosome-positive patients, the regimen has been further intensified to a “TKI + Venetoclax + chemotherapy + sequential CAR-T″ combination (NCT06481228). Additionally, for high-risk B-ALL, studies are exploring the synergistic effects of Venetoclax combined with hypomethylating agents and CD19/CD22 dual-target CAR-T (NCT06078306). These investigations collectively aim to enhance tumor cell apoptosis through Venetoclax, complementing the cytotoxic effects of CAR-T to achieve deep tumor clearance and improved outcomes. Concurrently, foundational research is progressing, with projects specifically examining the impact of targeted therapies on CAR-T cell generation capacity in CLL patients, providing theoretical support for optimizing combination strategies (NCT04640909). Overall, the field is transitioning from mere efficacy validation toward the construction of precise and personalized combination paradigms.

On the technological front, innovative delivery platforms are being explored to mitigate systemic toxicity and improve efficacy, such as a mucoadhesive oral carrier for the co-delivery of navitoclax and BCL2-targeting siRNA, which has demonstrated enhanced local drug retention and superior anti-tumor activity in preclinical models.

Looking forward, the future of integrating BCL2 inhibition with immunomodulation will hinge on the development of more precise and potent combinatorial regimens. Next-generation strategies will likely involve triple or quadruple combinations, incorporating BCL2 inhibitors into regimens containing CAR-T cells, bispecific antibodies, or therapeutic cancer vaccines (Liegel et al., 2021). The simultaneous targeting of additional immune checkpoints, such as CTLA-4, LAG-3, or TIGIT, may be required to fully overcome the immunosuppressive network of the tumor microenvironment. The development of more selective agents, including inhibitors targeting other BCL2 family members (e.g., BCL-XL, MCL-1), could help overcome resistance and provide a tailored approach that spares essential immune cells. Parallel to this, a major focus must be on biomarker-driven patient stratification. Comprehensive profiling of the tumor immune microenvironment, utilizing techniques like single-cell RNA sequencing and multiplex immunohistochemistry, will be crucial for identifying the patients who will derive the greatest benefit.

## 5 Discussion and outlook

### 5.1 Summary and cross-comparisons

The therapeutic landscape for hematologic malignancies has evolved dramatically over the past decade, shifting from conventional chemotherapy to targeted and immune-based strategies. BCL2 inhibitors, exemplified by venetoclax, have demonstrated profound efficacy in inducing apoptosis in malignancies dependent on BCL2 for survival, such as CLL and AML. However, resistance remains a formidable barrier, driven by compensatory upregulation of alternative anti-apoptotic proteins (e.g., MCL-1, BCL-XL), metabolic adaptations, and protective tumor microenvironment signals.

In parallel, immunomodulatory approaches—including IMiDs, CAR-T cells, BiTEs, and NK cell therapies—have revolutionized treatment by harnessing the immune system to recognize and eliminate malignant cells. These modalities achieve remarkable response rates in relapsed/refractory settings but are limited by antigen escape, T-cell exhaustion, and immunosuppressive microenvironments.

Cross-comparison reveals complementary strengths: BCL2 inhibitors directly target tumor-intrinsic survival pathways, while immunotherapies enhance extrinsic immune surveillance. Integrated strategies combining both modalities—such as venetoclax with anti-CD20 antibodies, IMiDs, or CAR-T cells—demonstrate synergistic efficacy by simultaneously inducing apoptosis and enhancing immune recognition, leading to deeper and more durable responses. In the treatment of relapsed/refractory chronic lymphocytic leukemia (CLL), the fixed-duration, chemotherapy-free regimen of venetoclax plus rituximab (VenR) demonstrates a dual superiority. When compared to chemoimmunotherapy with bendamustine and rituximab (BR) in the pivotal MURANO study, VenR more than tripled the median progression-free survival (54.7 vs. 17.0 months) and reduced the risk of death by 47%. Furthermore, VenR’s efficacy surpasses that of venetoclax monotherapy, achieving a substantially higher median PFS (54.7 vs. ∼25 months) and inducing deep molecular responses, with 70.3% of patients achieving undetectable minimal residual disease (uMRD) compared to only 5% with monotherapy. These data collectively establish the VenR combination as a highly effective strategy, offering profound and durable disease control superior to both traditional chemoimmunotherapy and targeted monotherapy (Kater et al., 2025; Li et al., 2024).

### 5.2 Consensus, controversies, and unresolved issues

The combination of BCL2 inhibitors and immunotherapies has reshaped the treatment paradigm for multiple hematologic malignancies. In CLL and AML, BCL2 inhibitors combined with hypomethylating agents or anti-CD20 antibodies have become one of the gold standards for first-line therapy, significantly improving response rates and overall survival. Meanwhile, immunotherapies—particularly CAR-T cells and bispecific T-cell engagers—have demonstrated breakthrough efficacy in B-cell malignancies and multiple myeloma. Integrated strategies that combine the strengths of both approaches—such as exploring sequential or synchronous administration of BCL2 inhibitors with CAR-T or bispecific antibodies—show immense potential to surpass the efficacy of monotherapies. By simultaneously targeting the survival mechanisms of tumor cells and activating the host immune system, these strategies aim to fundamentally overcome resistance mechanisms, thereby delivering deeper and more durable clinical remissions for patients.

The combined therapeutic strategy of BCL2 inhibitors and immunotherapy faces multiple challenges in clinical translation. Specific immune cells (such as pDCs) are susceptible to unintended damage due to their dependence on BCL2 for survival, leading to “on-target, off-tumor” risks; tumor cells can develop resistance by upregulating alternative proteins like BCL-XL/MCL-1 or remodeling the immunosuppressive microenvironment; the combination therapy also confronts the compounding toxicities of tumor lysis syndrome, myelosuppression, and immune-related adverse events; furthermore, the absence of predictive biomarkers exacerbates clinical decision-making complexity by hindering the identification of optimal patient subgroups.

Based on current research progress, several key scientific questions in this field remain to be urgently addressed: First, the mechanisms of immune escape mediated by antigen loss and T-cell dysfunction following CAR-T therapy have not been fully elucidated; second, the impact of BCL2 inhibition on the functional regulation of specific immune subsets such as plasmacytoid dendritic cells and its effect on the formation of long-term immune memory require systematic evaluation; third, the establishment of a biomarker system capable of predicting responses to combination therapies remains pending, which severely restricts the implementation of personalized treatment strategies. Furthermore, several therapeutic strategy issues need clarification, including the optimal sequencing of BCL2 inhibitors and immunotherapy, balancing clinical benefits against myelosuppressive toxicity in BCL2/MCL-1 dual inhibition strategies, and defining precise application pathways for immunotherapy in specific populations such as acute myeloid leukemia and T-cell malignancies. Resolving these critical issues depends on in-depth mechanistic exploration and well-designed clinical trials to provide evidence-based support.

### 5.3 Future research directions

#### 5.3.1 AI for resistance prediction

Artificial intelligence serves as a powerful tool for integrating multi-omics functional data—including genomics, transcriptomics, proteomics, metabolomics, drug sensitivity testing, and real-time apoptotic thresholds revealed by dynamic BH3 profiling. Through machine and deep learning algorithms, AI identifies key patterns and synergistic biological pathways underlying drug resistance from these complex datasets.

Specifically, AI can analyze dynamic BH3 profiling data to quantify changes in apoptotic priming, thereby predicting initial responses to BH3 mimetics (e.g., venetoclax) and the risk of acquired resistance (Bhatt et al., 2020). By integrating high-dimensional functional data from single-cell sequencing and mass cytometry, AI helps identify rare resistant subpopulations (e.g., leukemia stem cells) and decipher their distinct signaling and metabolic traits, offering potential targets to overcome resistance. Furthermore, AI enables systematic prediction of resistant phenotypes by uncovering synergistic pathways—such as the link between BCL-2 dependency and metabolic reprogramming—through integrated analysis of gene expression, proteomic, and metabolomic profiles (Didi et al., 2024).

#### 5.3.2 Advanced drug delivery systems

The future of hematologic malignancy treatment relies on the intelligent development of advanced drug delivery systems. To address core challenges—including tumor heterogeneity, the protective bone marrow microenvironment, and treatment resistance—novel delivery platforms that combine targeting capability, stimulus-responsiveness, and combinatorial drug delivery are urgently needed (Pan and Mahato, 2025). Key research directions include: developing lipid- and polymer-based nanoparticle platforms for synergistic multi-drug therapy; engineering intelligent systems with active targeting and stimulus-responsive features to enable precise intervention at disease sites; constructing biomimetic 3D scaffolds as localized delivery platforms to effectively eliminate minimal residual disease; and integrating nanotechnology with gene editing and immunotherapy to advance next-generation gene delivery vectors and anticancer vaccines. The incorporation of artificial intelligence and other cutting-edge technologies will accelerate the optimization of drug delivery systems, driving the evolution of hematologic cancer treatment from conventional approaches toward precise, efficient, and intelligent therapeutic strategies.

#### 5.3.3 PROTACs for BCL2 degradation

PROTAC (Proteolysis-Targeting Chimera) technology represents a paradigm shift in targeted therapy from “inhibition” to “degradation.” Through its unique bifunctional molecular structure—with one end binding to the target protein (e.g., BCL-2) and the other recruiting an E3 ubiquitin ligase (e.g., VHL)—PROTAC directs the intracellular ubiquitin-proteasome system to irreversibly degrade the target protein, while itself acting as a recyclable catalyst. As a next-generation strategy, VHL-based PROTACs demonstrate significant advantages: the ability to simultaneously degrade multiple anti-apoptotic proteins such as BCL-2 and BCL-xL; avoidance of platelet toxicity associated with traditional BCL-xL inhibitors through tissue-selective design; and most importantly, the potential to overcome resistance to Venetoclax caused by mechanisms like BCL-2 mutations. This technology offers a highly promising new direction for breaking through the current resistance barriers in BCL-2-targeted therapies.

## 6 Conclusion

Recent years have witnessed significant breakthroughs in overcoming drug resistance in hematologic malignancies. BCL-2 inhibitors effectively address survival resistance caused by BCL-2 overexpression by reactivating tumor cell apoptosis pathways, while immunotherapies such as CAR-T and BiTE successfully overcome tumor immune evasion mechanisms by enhancing immune cell function.

The strategy combining BCL-2 inhibition with immunomodulation simultaneously targets two core resistance mechanisms: intrinsic tumor cell survival pathways and impaired immune surveillance, generating synergistic effects. BCL-2 inhibitor-induced immunogenic cell death provides targets for immune attacks, while the activated immune system effectively eliminates apoptosis-resistant tumor cells.

Future optimization of combination strategies may include utilizing artificial intelligence for resistance prediction, developing PROTACs protein degradation technology, and designing intelligent drug delivery systems. Through deepened mechanistic research and innovative clinical trials, we anticipate ushering in a new era of precise and synergistic therapy for hematologic malignancies.
